# The enteric nervous system promotes intestinal health by constraining microbiota composition

**DOI:** 10.1371/journal.pbio.2000689

**Published:** 2017-02-16

**Authors:** Annah S. Rolig, Erika K. Mittge, Julia Ganz, Josh V. Troll, Ellie Melancon, Travis J. Wiles, Kristin Alligood, W. Zac Stephens, Judith S. Eisen, Karen Guillemin

**Affiliations:** 1 Institute of Molecular Biology, University of Oregon, Eugene, Oregon, United States of America; 2 Institute of Neuroscience, University of Oregon, Eugene, Oregon, United States of America; 3 Humans and the Microbiome Program, Canadian Institute for Advanced Research, Toronto, Ontario, Canada; Massachusetts Institute of Technology, United States of America

## Abstract

Sustaining a balanced intestinal microbial community is critical for maintaining intestinal health and preventing chronic inflammation. The gut is a highly dynamic environment, subject to periodic waves of peristaltic activity. We hypothesized that this dynamic environment is a prerequisite for a balanced microbial community and that the enteric nervous system (ENS), a chief regulator of physiological processes within the gut, profoundly influences gut microbiota composition. We found that zebrafish lacking an ENS due to a mutation in the Hirschsprung disease gene, *sox10*, develop microbiota-dependent inflammation that is transmissible between hosts. Profiling microbial communities across a spectrum of inflammatory phenotypes revealed that increased levels of inflammation were linked to an overabundance of pro-inflammatory bacterial lineages and a lack of anti-inflammatory bacterial lineages. Moreover, either administering a representative anti-inflammatory strain or restoring ENS function corrected the pathology. Thus, we demonstrate that the ENS modulates gut microbiota community membership to maintain intestinal health.

## Author summary

Intestinal health depends on maintaining a balanced microbial community within the highly dynamic environment of the intestine. Every few minutes, this environment is rocked by peristaltic waves of muscular contraction and relaxation through a process regulated by the enteric nervous system (ENS). We hypothesized that normal, healthy intestinal microbial communities are adapted to this dynamic environment, and that their composition would become perturbed without a functional ENS. To test this idea, we used a model organism, the zebrafish, with a genetic mutation that prevents formation of the ENS. We found that some mutant individuals without an ENS develop high levels of inflammation, whereas other mutants have normal intestines. We profiled the intestinal bacteria of inflamed and healthy mutants and found that the intestines of inflamed individuals have an overabundance of pro-inflammatory bacterial lineages, lack anti-inflammatory bacterial lineages, and are able to transmit inflammation to individuals with a normally functioning ENS. Conversely, we were able to prevent inflammation in the ENS mutants by either administering a representative anti-inflammatory bacterial strain or restoring ENS function. From these experiments, we conclude that the ENS modulates intestinal microbiota community membership to maintain intestinal health.

## Introduction

The intestinal tract serves to harvest nutrients and energy, protect against harmful toxins and pathogens, and clear out waste. These functions can be modulated by both the enteric nervous system (ENS) and the trillions of symbiotic bacteria that reside within the gut [1–3]. Importantly, the influence of microbiota on intestinal functions and health depends on the constituent microbes. Alterations in microbial composition from those observed in “healthy” subjects are often defined as “dysbiotic,” which refers to communities that become perturbed in their composition such that they acquire pathogenic properties [4–6]. Given that the composition of the microbiota is critical for host health, it is significant that the intestinal microbial community is generally stable despite the highly dynamic internal environment of the intestinal tract [7,8], which experiences disruptions such as influxes of ingested matter, host secretion and epithelial cell turnover, and coordinated outward flow of material. How microbial community stability is achieved amid these constant perturbations is unknown. Hosts with impaired intestinal motility can develop dysbiosis and intestinal pathology [9,10], which suggests a profound role for the ENS in constraining microbiota composition. Here, we explore how the ENS shapes the ecology of the intestine, and we address key questions about the assembly of dysbiotic microbial communities, their functional properties, and strategies for their treatment—three aspects of dysbiosis that have been challenging to address from observational studies in humans. Our analysis reveals how, without ENS constraint, imbalances in pro- and anti-inflammatory members of the microbiota can drive intestinal pathology.

The most severe example of ENS dysfunction in humans is Hirschsprung disease (HSCR), an enteric neuropathy that results from a failure of neural crest–derived cells to form the distal ENS [3]. Approximately 30% of HSCR patients develop a severe form of intestinal dysbiosis, known as Hirschsprung-associated enterocolitis (HAEC) [9–11], which is distinguished by diarrhea, distension, fever, and, in extreme cases, sepsis and death [12]. Studies suggest that the etiology of HAEC has a microbial component, as both pathogenic bacteria [13] and alterations in commensal communities [9,10] have been linked to HAEC. Interestingly, patients with a broad range of human diseases, such as inflammatory bowel disease (IBD), cystic fibrosis [14], diabetes [15], malnutrition [16], and myotonic muscular dystrophy [17,18], also experience debilitating gastrointestinal (GI) symptoms. Although cause and effect are difficult to determine, these diseases are associated with both small intestinal bacterial overgrowth, a clinical syndrome often seen with impaired intestinal motility, and an altered microbiota, suggesting that impaired ENS function could be a driver of dysbiosis.

To explore how the ENS may prevent dysbiosis by constraining microbial populations, we turned to a zebrafish model of HSCR. Multiple well-described zebrafish lines carry mutations in HSCR loci [19–22]. The most extreme ENS loss is seen in mutants homozygous for a null mutation in the HSCR gene *sox10* [23,24]; these mutants entirely lack an ENS [24]. The mutant allele *t3* (*sox10*^*t3*^) homozygotes have diminished rhythmic peristaltic activity [21], making this an ideal model for dissecting the role of the ENS in host–microbe interactions. Zebrafish are well suited for examining ENS contributions to microbiota composition because we can monitor ENS development, absolute bacterial abundance, and disease phenotypes, such as neutrophil accumulation, across the entire intestine of individual larvae. Thus, we can assess system-level functional readouts that describe properties of the associated microbiota. Furthermore, the high fecundity and ease of working with zebrafish provide us with large sample sizes to increase the power of our experiments such that we can monitor how natural microbiota variation at the species level drives phenotypic variation.

In this study, we demonstrate that the ENS constrains the abundance and composition of the microbiota. We find that loss of the ENS in *sox10*^*t3*^ mutants results in assembly of a dysbiotic community leading to a microbe-driven intestinal inflammation that varies among individuals and resembles HAEC. Microbiota profiling across the spectrum of inflammatory states revealed that extreme intestinal inflammation is linked to an outgrowth of pro-inflammatory bacterial lineages and a reduction of anti-inflammatory bacterial lineages. Moreover, administering representative anti-inflammatory bacterial strains or transplanting wild-type (WT) ENS precursors to restore a WT ENS corrects the pathology in *sox10*^*t3*^ mutant hosts. Our analysis reveals that ENS function is a key feature of intestinal health that constrains the composition of the resident microbiota and prevents overgrowth of bacterial lineages that can drive disease.

## Results

### Loss of *sox10* results in intestinal bacterial overgrowth

**Fig 1:**
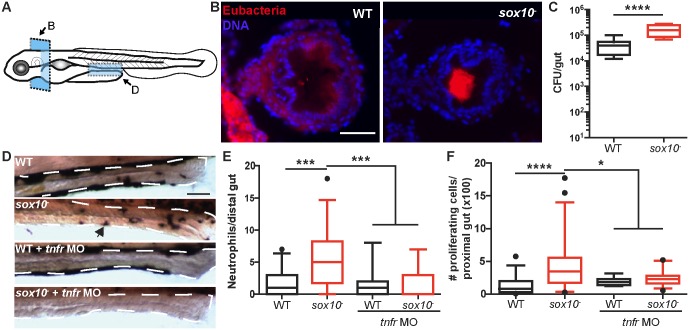
*sox10* mutants experience bacterial overgrowth and physiological indications of dysbiosis. **(A)** Schematic representation of the location and orientation of images in B and D. **(B)** Representative images of the panbacterial population by FISH on the esophageal-intestinal junction of WT (left) and *sox10*^*-*^ (right) fish. Blue, DNA; red, eubacteria. **(C)** Quantification of bacterial colonization level in *sox10* mutants and WT siblings. **(D)** Representative images of WT, *sox10* mutant, and tumor necrosis factor receptor (*tnfr*) morpholino (MO) injected larvae of both genotypes. Arrowhead indicates neutrophil. **(E)** Quantification of intestinal neutrophil number per 140 μm of distal intestine. **(F)** Total numbers of proliferating cells over 30 serial sections beginning at the esophageal-intestinal junction and proceeding into the bulb in 6-d-post-fertilization (dpf) fish. Box plots represent the median and interquartile range; whiskers represent the 5–95 percentile. *n* > 15 per group, *p < 0.05, ***p < 0.001, ****p < 0.0001, ANOVA with Tukey’s range test. Also see S1 Fig. Scale bars = 50 μm.

The complete loss of ENS in *sox10*^*t3*^ mutants (S1 Fig) results in defective intestinal motility [21]. Given the connection between altered intestinal motility and small intestinal bacterial overgrowth, we hypothesized that functional consequences of these mutants would include changes to intestinal ecology and alterations in resident microbial populations. To visualize the abundance and distribution of bacteria along the length of the intestine, we used fluorescent in situ hybridization (FISH). In *sox10*^*t3*^ mutants, we noted large populations of bacteria throughout the intestine, with marked accumulations of bacteria at the esophageal-intestinal junction (Fig 1A and 1B), a location not typically heavily colonized with bacteria. We also quantified the total number of colony-forming units (CFU) per intestine and found that *sox10*^*t3*^ mutants had a significantly higher bacterial load (Fig 1C). These results suggest that *sox10*^*t3*^ mutants experience bacterial overgrowth, which is consistent with defective intestinal transit. Defective intestinal transit has been observed in mutants in another allele, *sox10*^*m241*^, which have intestinal peristalsis but do not clear ingested fluorescent beads as well as WTs [25]. To demonstrate delayed intestinal transit in *sox10*^*t3*^ mutants, we adapted a previous single color assay [26] into a two-color intestinal transit assay (S1 Fig). The delayed transit and impaired clearance we observed in *sox10* mutants likely contribute to bacterial overgrowth within their intestines. For the work described in this manuscript, we use *sox10*^*t3*^ mutants, hereafter referred to as *sox10* mutant or *sox10*^*-*^.

### The *sox10*^*-*^ intestine exhibits increased neutrophil response and epithelial cell proliferation

We next asked whether the bacterial overgrowth phenotype in *sox10*^*-*^ resulted in signs of intestinal inflammation. Thus, we quantified intestinal neutrophil populations, a marker of inflammation, in cohoused WTs and *sox10* mutants by staining for the neutrophil-specific enzyme myeloid peroxidase. At 6 d post fertilization (dpf), intestinal neutrophil accumulation in *sox10* mutants was significantly increased compared to WTs (Fig 1D and 1E). Notably, *sox10* mutants exhibited a much greater variation in intestinal neutrophil accumulation (0–18; *n* = 30) compared to WT siblings (0–7; *n* = 31); some *sox10* mutants had intestinal neutrophil levels similar to WTs, whereas others had significantly elevated neutrophil populations. Intestinal neutrophil accumulation under homeostatic conditions in WT fish requires the pro-inflammatory tumor necrosis factor (TNF) pathway [27,28]. The increased neutrophil response in *sox10* mutants also depends on this pathway, as inhibiting expression of the TNF receptor using an antisense morpholino [27,28] abolished the increased neutrophil response (Fig 1D and 1E). Another indicator of intestinal pathology is epithelial cell proliferation. At 6 dpf, *sox10* mutants had markedly increased intestinal cell proliferation relative to cohoused WT animals. Unlike the normal intestinal epithelial cell proliferation response to microbiota, which is TNF independent [29], we found that elevated cell proliferation in the *sox10* mutant intestine was TNF dependent (Fig 1F), suggesting that this was an inflammation-dependent pathological response.

### The intestinal microbiota of *sox10* mutants is necessary and sufficient to induce a hyper-inflammatory state

**Fig 2:**
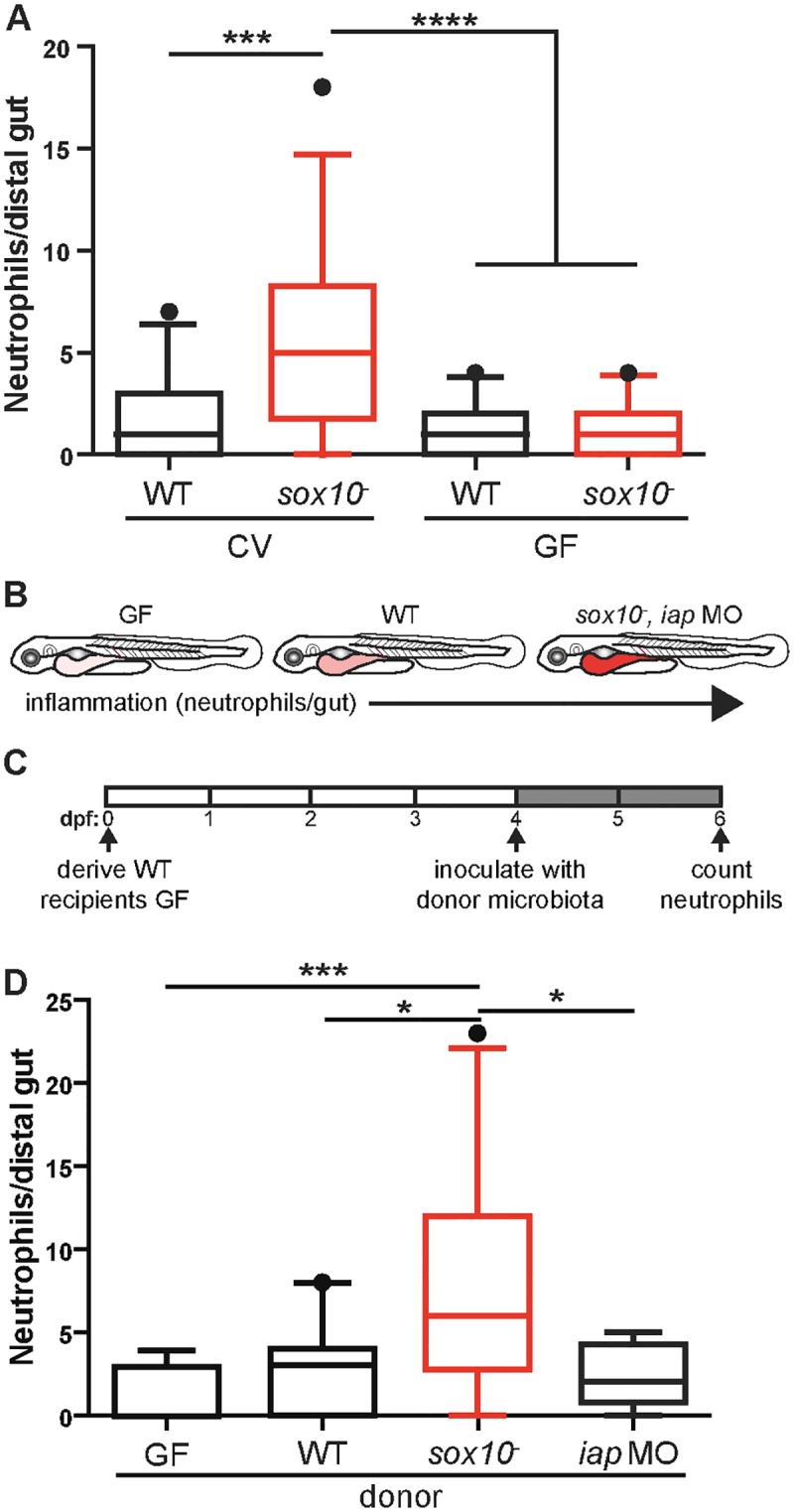
Intestinal microbiota are necessary and sufficient to induce increased intestinal neutrophil accumulation in *sox10* mutants. **(A)** Quantification of intestinal neutrophil number per 140 μm of distal intestine. Neutrophil accumulation was inhibited when *sox10* mutants were raised GF compared to CV controls. *n* > 21 per condition. **(B)** Schematic of fish used as donors in the transmission experiment. Intensity of red indicates level of intestinal inflammation. **(C)** Schematic of the experimental protocol. Intestines of GF, CV WT, *sox10* mutants, or *iap* MO were dissected for use as inoculum for 4 dpf GF WT recipients. Recipient fish were colonized for 2 d before examination of intestinal neutrophil number. **(D)** Transfer of intestinal microbes from inflamed intestines of *sox10* mutants causes increased intestinal neutrophil number in WTs. *n* ≥ 10, *p < 0.05, ***p < 0.001, ****p < 0.0001, ANOVA with Tukey’s range test. See also S2 Fig.

To determine whether the intestinal microbiota of *sox10*^*-*^ hosts is necessary to induce the increased intestinal neutrophil response, we derived *sox10* mutants and their WT siblings germ free (GF). We found that GF *sox10* mutants have a low neutrophil population, indistinguishable from their WT siblings (Fig 2A). To determine if the microbial community established in *sox10* mutants is sufficient to induce inflammation, we performed an experiment in which we transferred microbiota from *sox10* mutants into WTs. As donors, we used microbial communities from conventionally raised (CV) WT, *sox10* mutant, or WT intestinal alkaline phosphatase morpholino (*iap* MO)-injected larvae. *iap* MO-injected fish are hypersensitive to lipopolysaccharide and thus develop elevated intestinal inflammation without evidence of dysbiosis [27]. These fish serve as control for the possibility that nonbacterial factors such as host pro-inflammatory cytokines rather than microbial derived factors cause transmissible intestinal inflammation (Fig 2B) [30]. At 6 dpf, for each separate group (WT, *sox10*^*-*^, and *iap* MO), we dissected, pooled, and homogenized the donor intestines. As a negative control, we included transplantation from homogenized intestines of GF fish. The homogenate from each group was inoculated into flasks housing GF 4 dpf WT fish (Fig 2C). We found that inoculation with microbes from *sox10* mutants was sufficient to induce elevated intestinal inflammation in WTs as compared to inocula from GF, CV WT, or CV *iap* MO fish, none of which induced intestinal inflammation (Fig 2D). To test whether the capacity of *sox10* mutant microbiota to induce elevated neutrophils was due to increased bacterial load, we transplanted 5× CV WT microbes, which corresponded to the bacterial load of *sox10* mutant transplants. This larger inoculum did not induce more intestinal inflammation (S2 Fig), which indicates that the microbial community assembled in *sox10*^*-*^ hosts is functionally distinct from WT microbiota and is sufficient to induce inflammation in fish with a normal, functional ENS.

### Bacterial overgrowth does not explain increased intestinal neutrophil response

**Fig 3:**
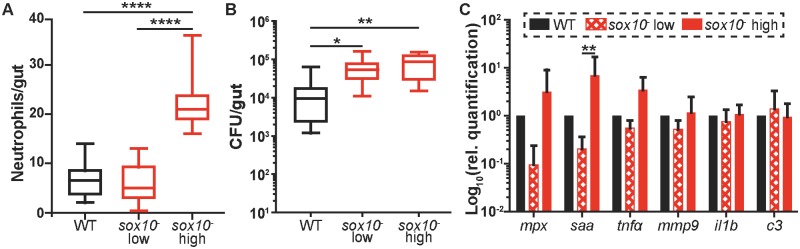
Increased bacterial colonization level does not drive increased intestinal neutrophil accumulation or pro-inflammatory gene expression. Quantification of intestinal neutrophil number **(A)** and bacterial colonization level **(B)** in the *sox10*^*-*^, Tg*(mpx*:*GFP)* line. *sox10*^*-*^ fish were split into two groups, “*sox10*^*-*^ low” (bottom half) and “*sox10*^*-*^ high” (top half) based on intestinal neutrophil number. Ten representative fish from each group were plated to determine total CFU/intestine. *n* ≥ 9 per group. *p < 0.05, **p < 0.01, ****p < 0.0001, ANOVA with Tukey’s range test. **(C)** Relative expression calculated by the 2^-ΔΔCt^ method of immune genes from dissected intestines. For *mpx*, *saa*, *il1b*, and *c3*, *n* = 5 pools of 5 dissected intestines; for *tnfα* and *mmp9*, *n* = 3 pools of 18 dissected intestines. Graph displays average ± standard deviation (SD); **p < 0.01, *t* test corrected for multiple comparisons using Holm–Šidák method.

*sox10* mutants exhibit a wide range of intestinal neutrophil populations (Figs 1D and 2A) as well as variation in bacterial load (Fig 1B). Therefore, we asked whether intestinal neutrophil abundance corresponded to increased bacterial abundance. We used transgenic *sox10* mutant hosts expressing green fluorescent protein (GFP) under control of the neutrophil-specific *mpx* promoter to quantify both neutrophil population and intestinal bacterial load in individual fish (Fig 3A). When we compared *sox10* mutants that fell in the bottom half of neutrophil response (“*sox10*^*-*^ low”) or in the top half of neutrophil response (“*sox10*^*-*^ high”) to WTs, we found that all *sox10* mutants, regardless of neutrophil level, carried significantly higher bacterial loads than WTs (Fig 3B). Thus, impaired intestinal clearance (S1 Fig) leads to an increased bacterial load; however, the bacterial overgrowth per se in *sox10*^*-*^ does not drive an increased intestinal neutrophil response. We further characterized the pro-inflammatory signature of the *sox10*^*-*^ high- and low-neutrophil subsets by monitoring expression of a panel of immune genes in the intestine (Fig 3C). These results aligned with our observations of the neutrophil population, as the *sox10*^*-*^ high-neutrophil subset had elevated levels of *mpx*, *saa*, and *tnfα* expression compared to WT and the *sox10*^*-*^ low-neutrophil subset (Fig 3C); however, the increase in *saa* transcription was the only one to reach statistical significance. Consistent with the significantly elevated intestinal neutrophil response in these samples, *saa* is known to mediate intestinal neutrophil behavior stimulated by microbes [31]. Collectively, our results suggest that a pro-inflammatory compositional change occurs in the microbial community of a subset of *sox10* mutants.

### Changes in the abundance of two dominant microbial genera drive intestinal neutrophil accumulation

**Fig 4:**
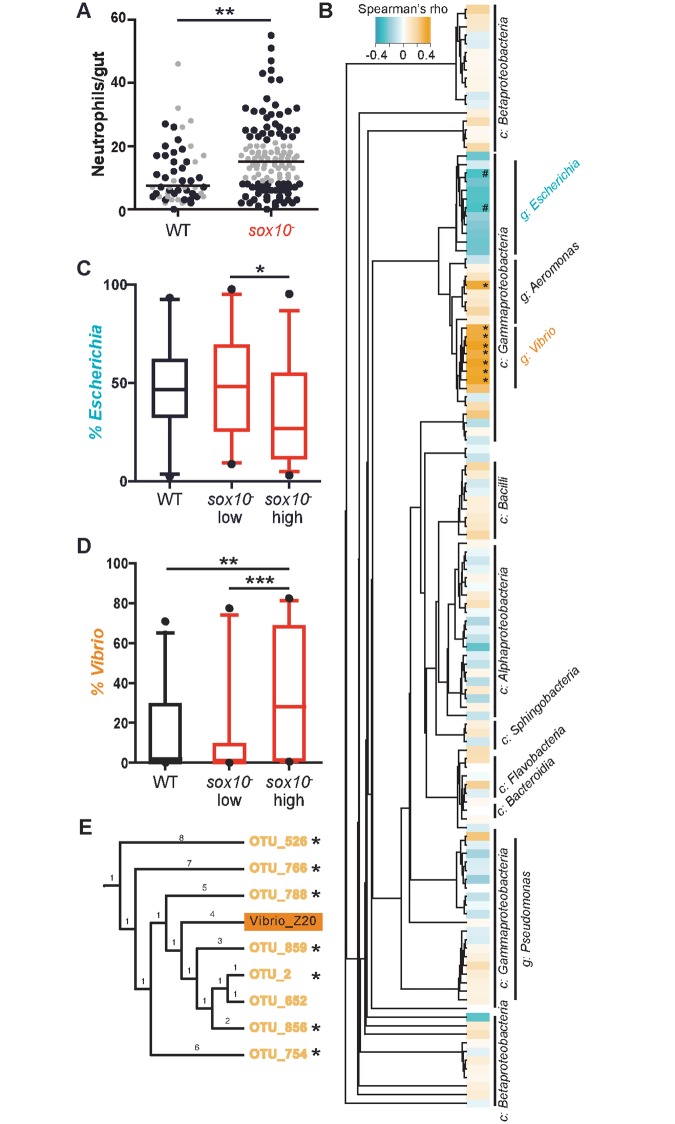
Changes in microbial lineages define low and high neutrophil accumulation. **(A)** Intestinal neutrophil accumulation from samples used for 16S rRNA gene sequencing. Each dot represents an individual fish; black circles indicate sequenced samples, WT, *n* = 32; *sox10*^*-*^ high, *n* = 30; *sox10*^*-*^ low, *n* = 31. Gray circles indicate samples that were not sequenced. Line indicates median. **p < 0.01 Student’s *t* test. **(B)** Spearman’s rank correlation between intestinal neutrophil number and each operational taxonomic unit (OTU) present in at least 20 samples. After false discovery rate correction, two genera, *Escherichia* and *Vibrio*, stand out with correlations to neutrophil number. Asterisks represent significance of Spearman correlation, *p < 0.05, #*p* = 0.08, c: class, g: genus. The percent abundance of *Escherichia* **(C)** and *Vibrio* **(D)** across genotypes and intestinal neutrophil levels. **(E)** Phylogenetic tree of OTUs from the *Vibrio* genus and our *Vibrio* zebrafish isolate (Vibrio Z20). For D, E: *p < 0.05, **p < 0.01, ***p < 0.001, ANOVA. See also S3 Fig.

To address the possibility of a pro-inflammatory compositional change in the *sox10*^*-*^ microbiota, we profiled microbial communities by performing 16S rRNA gene sequencing on intestinal communities isolated from cohoused WT and *sox10* mutant individuals. We collected samples across three independent experiments. To uncover differences in microbiota composition that explain the variable severity of neutrophil accumulation in *sox10* mutants, we collected intestinal neutrophil response data for the same individuals from which we isolated microbial DNA and grouped samples as “WT,” “*sox10*^*-*^ low” (intestinal neutrophil response 0–8), or “*sox10*^*-*^ high” (intestinal neutrophil response of greater than or equal to 22); these groups include the top 26% and the bottom 29%, respectively (Fig 4A). By standard metrics of community variability (non-metric multidimensional scaling of Canberra distances, richness, Faith’s Phylogenetic Diversity, unweighted UniFrac), these three groups were not significantly different (S3 Fig), which indicates that these communities are largely made up of the same microbes, and community differences driving neutrophil differences are perhaps due to changes in minor members [28].

We next asked whether the relative abundance of any bacterial operational taxonomic units (OTUs) correlated with intestinal neutrophil number across all individuals surveyed in the study. Of 129 OTUs present in at least 20 individuals, we found a small subset whose percent abundance was associated with neutrophil number, as measured by Spearman’s correlations. Strikingly, these neutrophil-associated OTUs were tightly clustered in only two genera found in this population of fish intestines (Fig 4B). The *Escherichia/Shigella* genus (hereafter referred to as *Escherichia)* had ten OTUs that negatively correlated with neutrophil abundance, although only two had borderline significance after false discovery rate correction. All OTUs of the *Vibrio* genus had significant positive correlations with neutrophil abundance (Fig 4B). Examination of OTU abundances revealed not only that the two most abundant genera were *Vibrio* and *Escherichia* (S3 Fig) but also that they were significantly decreased and increased, respectively, in the “*sox10*^*-*^ high” group relative to the WT and “*sox10*^-^ low” groups (Fig 4C and 4D).

**Fig 5:**
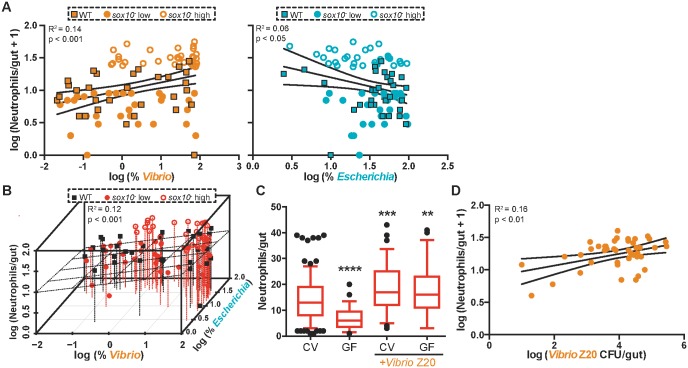
An increase in *Vibrio* contributes dominantly to intestinal neutrophil number. **(A)** Correlation between the log percent *Vibrio* abundance (left) and log percent *Escherichia* abundance (right) with log_10_(intestinal neutrophil number +1). Linear regression analysis with 95% confidence intervals. Each point represents an individual fish: WT, squares, *n* = 32; *sox10*^*-*^ high, open circles, *n* = 30; *sox10*^*-*^ low, closed circles, *n* = 31. **(B)** Correlation between both log_10_(percent *Escherichia*) (*z*-axis) and log_10_(percent *Vibrio*) (*x*-axis) abundance with log_10_(intestinal neutrophil number +1) (*y*-axis). Planar regression analysis, *n* = 93. WT, black squares; *sox10*^*-*^ low, red closed circles; *sox10*^*-*^ high, red open circles. **(C)** Addition of *Vibrio* Z20 increased intestinal neutrophil accumulation in both CV and GF *sox10* mutants. *n* ≥ 49, from at least three independent experiments. **p < 0.01, ***p < 0.001, ****p < 0.0001, indicates difference from CV, ANOVA. **(D)** Correlation between the absolute abundance of *Vibrio* Z20 in CV *sox10* mutants and log_10_(intestinal neutrophil number + 1) in experiments with exogenously added *Vibrio* Z20. Linear regression analysis with 95% confidence intervals. For C-D, data from four to six independent experiments, *n* > 35. See also S4 Fig.

The observation of pro-inflammatory activity associated with *Vibrio* is consistent with our previous analysis of a zebrafish-derived *Vibrio* strain (ZWU0020) [32] that is phylogenetically closely related to the *Vibrio* OTUs in the current experiment (Fig 4E). Previously, we showed that *Vibrio* strain ZWU0020 (hereafter referred to as *Vibrio* Z20) promotes intestinal neutrophil accumulation in a concentration-dependent manner in gnotobiotic zebrafish [28]. Similarly, in the current study, the log_10_(relative abundance) of *Vibrio* was significantly positively correlated with neutrophil number (Fig 5A), and we also observed that the log_10_(relative abundance) of *Escherichia* was negatively correlated with intestinal neutrophil accumulation, although the amount of variation explained was low (Fig 5A). This relationship mirrors the relationship we previously observed in simple microbial communities in gnotobiotic zebrafish between the abundance of *Shewanella* strain ZOR0012 (hereafter referred to as *Shewanella* Z12) and a proportional decrease in neutrophil number [28]. Of note, two OTUs from the *Shewanella* genus included in our analysis in this study did not have a significant correlation with neutrophil number. Combining the loss of *Escherichia* and the gain of *Vibrio* does not increase the amount of variation in neutrophil number explained by the gain of *Vibrio* alone (Table 1, Fig 5B). We used Akaike’s Information Criterion (AIC) [33] to test the relative quality of each of these models; the model that accounts only for *Vibrio* reports the lowest AIC value, which identifies *Vibrio* as the best microbial predictor of intestinal neutrophil number variability (Table 1). These analyses suggest that a balance of the *Vibrio* and *Escherichia* lineages may be important for maintaining intestinal homeostasis, with *Vibrio* abundance being a major determinant of intestinal inflammation.

**Table 1:** Coefficients of regression analysis of *Escherichia*, *Vibrio*, and intestinal neutrophil number.

		Estimate	Std. err.	*t*-value	*p*-value	MultipleR^2^	*p*-value	A.I.C.	AIC relative likelihood
***Escherichia***	(Intercept)	1.48	0.18	8.0	<0.0001	0.06	0.02	93.5	0.04
	*Escherichia*	−12.5	3.7	−3.3	0.001				
***Vibrio***	(Intercept)	0.99	0.043	23.5	<0.0001	0.14	0.0003	87.1	1
	*Vibrio*	0.12	0.035	3.5	0.0007				
***Escherichia* and *Vibrio***	(Intercept)	1.20	0.21	5.91	<0.0001	0.13	0.0019	88.1	0.6
	*Escherichia*	−0.13	0.13	−1.03	0.31				
	*Vibrio*	0.11	0.04	2.73	0.0074				

To confirm the functional contribution of *Vibrio* to the increased neutrophil responses in *sox10*^*-*^, we first added *Vibrio* Z20 to CV *sox10* mutants at 4 dpf and assayed neutrophil numbers at 6 dpf. Exogenously added *Vibrio* Z20 induced a significant increase in neutrophil accumulation over the number seen in CV *sox10* mutants (Fig 5C). Furthermore, the absolute abundance of *Vibrio* Z20 colonizing these fish was positively correlated with intestinal neutrophil number, similar to observations made in the 16S rRNA data set (Fig 5D). We noted that the extent of the increase in neutrophil accumulation upon addition of *Vibrio* depended on the level of intestinal neutrophils present in control hosts (S4 Fig), which we think reflects fluctuations in bacterial community composition in CV zebrafish between experiments and a limited ability to change the neutrophil-inducing capacity of an intestinal microbiota already dominated by *Vibrio* strains. We furthermore observed the inflammation-inducing capacity of *Vibrio* Z20 in monoassociation by adding *Vibrio* Z20 to GF *sox10* mutants. In these conditions, Vibrio Z20 was still sufficient to induce high intestinal neutrophil influx (Fig 5C). In monoassociation the range of *Vibrio* colonization was too narrow (S4 Fig) to explore a correlative relationship. These experiments support our hypothesis, based on the microbiota profiling of these fish, that an overabundance of *Vibrio* species causes a dysbiotic and pro-inflammatory microbiota.

### Intestinal hyper-inflammation is ameliorated by anti-inflammatory bacterial isolates or by transplantation of WT ENS precursors into sox10 mutants

**Fig 6:**
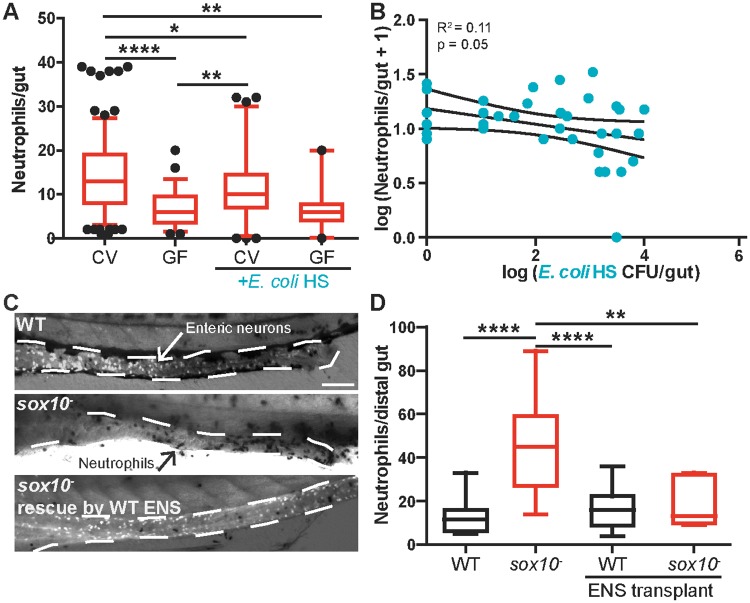
Inflamed intestines are rescued by anti-inflammatory bacterial isolates or transplantation of WT ENS into *sox10* mutants. **(A)** Addition of a representative *Escherichia* isolate, *E*. *coli* HS, to CV *sox10* mutants reduces intestinal neutrophil accumulation. Monoassociation of *sox10* mutants with *E*. *coli* HS does not increase neutrophil level over that observed in GF zebrafish. *n* > 20, from at least three independent experiments. **(B)** Correlation between absolute abundance of *E*. *coli* HS and log_10_(intestinal neutrophil number + 1) in experiments with added *E*. *coli* HS. Linear regression analysis with 95% confidence intervals. For A, B: *n* > 35, from three to six independent experiments. **(C)** Representative images of distal intestine from WT, *sox10*^*-*^, and *sox10*^*-*^ rescued by WT ENS precursor transplantation. Anti-ElavI1–labeled enteric neurons are white (white arrow); neutrophils are black (black arrow). Scale bar = 100 μm. **(D)** Quantification of intestinal neutrophil number per 140 μm of distal intestine. *n* > 6 for all conditions, *p < 0.05, **p < 0.01, ****p < 0.0001, ANOVA with Tukey’s range test. See also S4 Fig.

We hypothesized that the dysbiotic state of the *sox10* mutant intestine could be corrected by balancing the pro-inflammatory activity of *Vibrio* species with the addition of anti-inflammatory isolates, such as *Escherichia* species or *Shewanella* Z12 [28]. Consistent with this prediction, we found that addition of *Escherichia coli* HS, a commensal *Escherichia* strain isolated from a healthy human adult [34] that is closely related to *Escherichia* OTUs in CV fish (S4 Fig), can colonize the zebrafish intestine (S4 Fig), reducing neutrophil numbers in CV *sox10* mutants (Fig 6A) and maintaining GF levels of intestinal neutrophil accumulation in monoassociation (Fig 6A). Moreover, the absolute abundance of colonizing *E*. *coli* HS in CV *sox10* mutants displayed a similar negative correlation with intestinal neutrophils (Fig 6B), as observed with the sequenced OTUs (Fig 5A). *Shewanella* Z12, another species that displays a negative correlation between abundance and intestinal neutrophil accumulation [28], also reduced intestinal neutrophils in *sox10* mutants, which suggests this relationship may be a hallmark of anti-inflammatory bacterial strains (S4 Fig). Thus, *sox10*^*-*^ dysbiosis can be corrected by adding anti-inflammatory bacteria to the community. *Shewanella* Z12 uses an unidentified secreted factor present in cell-free supernatant (CFS) to mediate its anti-inflammatory activity [28] (S4 Fig). However, *E*. *coli* HS CFS was insufficient to reduce *sox10* mutant intestinal inflammation (S4 Fig), suggesting that these species use two distinct mechanisms to control the host innate immune response.

As an alternative to manipulating the microbiota directly, we postulated that correcting the underlying deficit in the ENS would also alleviate the inflammation in *sox10* mutants. To test this hypothesis, we performed a rescue experiment in which vagal neural crest cells, the ENS precursors, were transplanted from WT donors into *sox10* mutant hosts. Our previous studies showed a correlation between the number of ENS neurons and gut motility [21]; thus, after the transplant, we assayed for formation of a normal-appearing ENS along with intestinal neutrophil accumulation. Following transplantation, the *sox10* mutants that developed a normal-appearing ENS extending along the entire length of the intestine had WT levels of intestinal neutrophils (Fig 6C and 6D), demonstrating that the ENS is sufficient to prevent intestinal inflammation. Together, our results demonstrate that the ENS contributes to intestinal health by maintaining a balanced gut microbiota, revealing a previously unappreciated role for the ENS in host–microbe interactions.

## Discussion

Many intestinal diseases, such as IBD, and many diseases with intestinal symptoms, such as cystic fibrosis [14], are associated with compositional changes in the intestinal microbiota, implying dysbiosis. However, it has been extremely challenging to establish whether such alterations are indeed dysbiotic and the underlying driver of disease. Overcoming this challenge will be an important step toward identifying new therapeutic targets and strategies for ameliorating dysbiosis-associated disease. To connect alterations in microbial communities with host pathology, three questions are crucial to address: (1) How do dysbiotic microbial communities assemble? (2) Which member(s) of dysbiotic communities contribute to disease? (3) How can dysbiosis-related disease be mitigated?

### How do dysbiotic microbial communities assemble?

**Fig 7:**
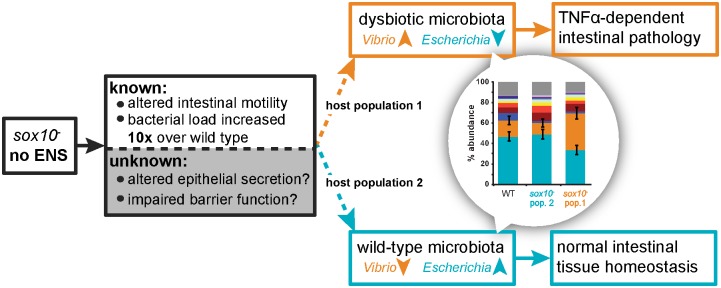
Proposed model of *sox10* mutant intestinal pathology. *sox10* mutants have altered intestinal motility and an increased bacterial load. Given the role of the ENS in intestinal function, *sox10* mutants likely also experience alterations in epithelial secretion and permeability, although these phenotypes are yet to be examined. *sox10* mutants can assemble a microbiota that mirrors WT intestinal microbiota (host population 2) or is dysbiotic (host population 1), characterized by an expansion of the *Vibrio* lineage and reduction of the *Escherichia* lineage. We do not yet know what determines which bacterial community assembles in *sox10* mutants (dashed lines) but hypothesize that it could be due to the timing or order of exposure to bacterial strains, differences in epithelial permeability or secretion, or differences in other host compensatory mechanisms.

Microbiota are assembled through fundamental ecological processes, including dispersal, local diversification, ecological drift, and environmental selection [35]. We have previously shown that a portion of early larval zebrafish intestinal communities follow a neutral pattern of assembly [36]. This observation suggests that features of the gut environment constrain which microbes colonize and persist in the gut environment. We hypothesized that the ENS, which controls motility and aspects of intestinal homeostasis [3], may also directly or indirectly serve as a significant constraint on intestinal microbial community assembly, such that loss of the ENS constitutes a major ecological shift. Consistent with this hypothesis, we show that zebrafish lacking an ENS have an altered intestinal microbiota and deficits in clearing food from the gut, suggesting gut motility is a mechanism by which the ENS influences microbiota composition. This is further supported by the recent finding that GI transit time is one of the largest predictors of microbiota composition [37]. Moreover, intestinal motility profoundly influences the spatial organization of bacterial populations and has been found to promote competitive exclusion within resident communities [38]. This suggests that abnormal GI transit patterns can significantly reshape ecological interactions within the gut. The ENS also contributes to epithelial barrier function and secretion; however, whether and how these functions are altered in the *sox10* mutant has not yet been described. Therefore, observed alterations to the microbial community may be the result of changes to any (or all) of these functions (Fig 7). Of all ENS mutants, the *sox10* mutant has the most overlapping characteristics with the human disease HSCR; however, given that the ENS is interconnected with many other organ systems, our work reveals the need to investigate other model systems of ENS dysfunction. Currently, no other available mutant both entirely eliminates the ENS as seen in *sox10* mutants and retains normal craniofacial structures [21]. For example, another severe mutant, *ret*, has a few residual ENS neurons and also exhibits severe craniofacial defects that may impair bacterial colonization [39]. A zebrafish *sox10* cell ablation model exists [40] but requires treatment with the antibiotic metronidazole, which would alter the microbiota and confound our experiments. For future experiments, developing a new line in which it is possible to specifically ablate enteric neurons at specified developmental stages will be essential.

Mounting evidence suggests that ENS defects of HSCR patients, as well as those of HSCR animal models, are not restricted to the aganglionic region of the intestine but rather extend to more proximal intestinal regions; thus, these defects are poised to precipitate dysbiosis associated with HAEC [9,41,42]. Patients with chronic IBD can also have functional and structural abnormalities in the ENS that disrupt motility [43,44]. Although these pathologies are generally thought to be secondary to inflammation [45], our data raise the possibility that, regardless of the origin of ENS defects, they have the potential to disrupt the microbial community and thus contribute to a feedback loop that prevents a healthy microbiota from establishing after an inflammatory episode. Such a cycle could explain the variable outcomes of treating IBD patients with probiotics [46]. However, in healthy hosts, this feedback loop may instead enforce stability and homeostasis within the system.

### Which member(s) of dysbiotic communities contribute to disease?

The intimate connection between human health and microbiota suggests that health is an effect of services provided by the microbial ecosystem [35], and thus to manage health through the microbiota, we need to identify the taxa that provide specific ecosystem services. One strategy to identify bacterial species that specifically influence host health or disease phenotypes is to define a dose–response relationship, or correlation, between a phenotype of interest and a microbial isolate. For example, we used gnotobiotic methods to identify *Vibrio* Z20 as pro-inflammatory in zebrafish by its positive correlation between abundance and intestinal neutrophil number [28], defining a dose–response relationship for this isolate. In this study, we have now expanded this approach to complex communities and discovered that *Vibrio* species fulfill this pro-inflammatory role in the highly inflamed *sox10* mutant gut—finding a positive relationship between relative abundance of *Vibrio* and number of intestinal neutrophils. Correlations between OTUs and host phenotypes have been important in the identification of “indicator” species of interest in chronic obstructive pulmonary disease [47], ulcerative colitis [48], and asthma [49]. Perez-Losada and colleagues [49] expanded this concept by comparing the host and bacterial transcriptomes of asthmatics and healthy controls. They revealed positive correlations between both bacterial phyla (Proteobacteria) and functions (adhesion) with the pro-inflammatory cytokine IL1A [49]. Similarly, a recent large-scale study used correlations to identify microbial drivers of cytokine expression in healthy humans [50]. These studies highlight the potential for using correlation to identify bacterial species, or properties of bacterial species, that have functional consequences for the host in health and disease.

The zebrafish is an especially good model for this type of analysis because we can manipulate host genetics and the environment to control microbial variability across samples. For human studies, the heterogeneity in microbial communities among subjects may be a limiting factor in performing this type of analysis. Furthermore, zebrafish microbial communities are less complex than those of humans, which allows us to probe the data at a higher resolution, with less data reduction [48], and to analyze host–microbe interactions at the OTU level. Our analysis at this resolution revealed conserved functions at the genera level. The phylogenetic conservation of certain bacterial traits suggests that interactions between zebrafish and their resident microbiota serve as a model for identifying bacterial lineages that influence phenotypes across many host species. For example, like the pro-inflammatory *Vibrio* identified in *sox10* mutants, some *Vibrios*, such as *Vibrio parahaemolyticus*, induce inflammatory gastroenteritis in humans [51]. We also identified the *Escherichia* genus as anti-inflammatory in the zebrafish, and some *Escherichia* are used as probiotics in the treatment of inflammatory intestinal disorders like ulcerative colitis [52]. These data suggest that characterizing species correlated with host phenotypes in model organisms may help to identify individual members of complex communities that contribute to disease phenotypes.

### How can dysbiosis-associated disease be mitigated?

Viewing host–microbiota interactions as an ecological system allowed us to identify two system components, the ENS and key bacterial species, which greatly influence ecosystem function, as measured by host intestinal inflammation. With this information, we can ask whether manipulation of these components provides us with control over ecosystem function. For example, an expanded population of *Vibrio* lineages combined with a decreased population of *Escherichia* lineages in *sox10* mutants induces increased neutrophil influx. We manipulated this component by introducing the anti-inflammatory *Escherichia* or *Shewanella* Z12 [28] and thus ameliorated the disease phenotype. Notably, the most consistent microbial signature of IBD patients is the loss of an anti-inflammatory species, *Faecalibacterium prausnitzii* [53], the colonization level of which decreases in a step-wise manner from healthy subjects, to patients in remission, to patients with active colitis, to patients with infective colitis [54]. Furthermore, administration of *F*. *prausnitzii* reduces disease severity in mice with chemically induced colitis [55]. These results highlight the important immunomodulatory role played by specific bacterial species within the intestinal microbiota and the need to identify these species to devise therapies for reestablishing control of the intestinal environment and ameliorating dysbiosis.

Treatment of dysbiosis-associated diseases with probiotics is likely to require continual probiotic administration if there is an underlying disease mechanism leading to its depletion. A more fruitful approach would include a treatment for the underlying ecological perturbation along with the introduction of probiotic strains. For example, restoring ENS function via transplantation or drug administration are possible ways to treat ENS dysfunction. We demonstrated that transplantation of WT ENS precursors into *sox10* mutant hosts restored a normal-appearing ENS and rescued the inflammatory gut phenotype. We think that the normal inflammatory response indicates restored ENS function. However, in future experiments, determining the functional capacity of the transplanted ENS to restore motility, secretion, and epithelial barrier function will help elucidate which specific ENS functions contribute to the constraint on the intestinal microbiota. Recently, there has been significant success in establishing a functional ENS in mouse by transplantation of induced pluripotent stem cells [56], which, together with our results, suggests that this strategy could contribute to a successful cure of disease in cases of HAEC or IBD.

## Conclusion

Here, we have utilized the zebrafish as a powerful model to examine the complex relationship between the ENS, the immune system, and the microbiota. We demonstrated the critical role played by the ENS in shaping the ecology of the intestine by constraining the functional properties of the resident microbiota. Our analysis reveals how, without this constraint, imbalances in pro- and anti-inflammatory members of the microbiota can drive intestinal pathology. The imbalances we discovered could not be described by large changes in phylum level abundances or the acquisition of a single pathogenic lineage but rather by subtle differences in the abundances of key commensal species that have the potential to either protect against or promote inflammation. We note that this discovery reveals the reciprocal relationship between the microbes and the ENS, as ENS activity and development can be altered by microbiota; in fact, individual bacterial species can have distinct effects on ENS function [57]. Furthermore, immune cell responses influence ENS function both under healthy conditions [58] and in inflammatory states [59]. Therefore, intestinal homeostasis depends on a complex tri-directional conversation that occurs between the microbiota, the ENS, and the immune system, with proper functioning of each branch depending on signals from the other two branches. Uncovering new therapeutic strategies for chronic intestinal diseases will require a profound understanding not only of each branch of this system but the multifaceted interactions that connect them and how alterations made to one system ripple out to affect the function of the other two branches. Developing scalable and tractable model systems, such as the zebrafish, in which we can monitor all three branches of this system will be critical for addressing these complex questions.

## Materials and methods

### Ethics statement

All zebrafish experiments were done in accordance with protocols approved by the University of Oregon Institutional Animal Care and Use Committee (protocol numbers 15–15, 14-14RR, and 15-83A8) and conducted following standard protocols as described in [60].

### Zebrafish husbandry

CV-raised WT (AB x Tu strain), heterozygote *sox10*^*t3-*^ (referred to as *sox10*^*-*^) [24], and *Tg(BACmpx*:*GFP)*^*i114*^ (referred to as *mpx*:*GFP*) [61] fish were maintained as described [60]. Homozygous *sox10* mutants were obtained by mating heterozygotes and identified by lack of pigmentation [24]. The *sox10*^*t3*^ line was used for neutrophil experiments unless otherwise indicated. The *mpx*:*GFP* line [61] was crossed with *sox10*^*+/-*^ adults to create a line that when in-crossed resulted in offspring that were *sox10*^*-/-*^ and *Tg(BACmpx*:*GFP)*^*i114*^ (referred to as *sox10*, *mpx*:*GFP*). No defects were observed in heterozygous siblings, which have pigment, develop normally, and survive to adulthood, and thus they are grouped with homozygous WTs [21,23,24,38,62]. For all experiments, WT siblings and homozygous *sox10* mutants were cohoused.

### Food transit assay and fluorescent in situ hybridization

See S1 Text.

### Morpholino injections

Splice-blocking MOs (Gene Tools, Corvallis, OR) were injected into embryos at the one cell stage. For knockdown of TNF, the *tr1v1*/*tr1v2* MOs (1.2 moles and 6 moles, respectively) were used as previously described [27,28]. For knockdown of intestinal alkaline phosphatase, the *iape212* MO (3 pmoles) was used as previously described [27].

### Histology and quantification of neutrophils and proliferating cells

Zebrafish larvae were fixed in 4% paraformaldehyde (PFA) overnight. Whole larvae were stained with Myeloperoxidase kit (Sigma) following the manufacturer’s protocol and processed and analyzed as previously described [27]. For analysis of neutrophils in *mpx*:*GFP* fish, GFP+ cells in the intestine were quantified as previously described [28]. For proliferation, larvae were immersed in 100 μg/ml EdU (A10044, Invitrogen) for 16 h prior to PFA fixation. Subsequent processing and analysis were done as previously described [29]. See also Histology and neutrophil analysis in S1 Text.

### Microbiota quantification

At 6 dpf, larvae were humanely killed with Tricaine (Western Chemical, Inc., Ferndale, WA), mounted in 4% methylcellulose (Fisher, Fair Lawn, NJ), and their intestines were dissected using sterile technique. Dissected zebrafish intestines were placed in 100-μl sterile EM, homogenized, diluted, and cultured on tryptic soy agar plates (TSA; BD, Sparks MD). After incubation at 32°C for 48 h for conventionally colonized fish or for 24 h for inoculated fish, colonies were counted.

### Gnotobiotic fish husbandry

Zebrafish embryos were derived GF as previously described [63]. All manipulations to the GF flasks were performed under a class II A/B3 biological safety cabinet. Zebrafish inoculated with donor microbial populations were generated by inoculating flasks with 4 dpf GF zebrafish with 10^4^ CFU/mL of donor microbes (1×). Donor microbes were collected by dissecting CV zebrafish and based on colonization data (Fig 1B) each fish was assumed to carry 10^5^ total CFU/gut; a total of 25 dissected guts were pooled and homogenized to create the donor microbes. We inoculated CV fish with live *Vibrio* (10^6^ bacterial cells/ml), *E*. *coli* HS (10^7^ bacterial cells/ml), and *Shewanella* Z12 (10^6^ bacterial cells/ml) as previously described [28]. For monoassociations, each strain was inoculated at 10^6^ bacterial cells/ml. We isolated and concentrated CFS as previously described [28]. Flasks were kept at 28°C until analysis of myeloperoxidase positive cells on 6 dpf.

### RNA isolation and qPCR

RNA isolation and cDNA preparation were performed as previously reported [27] except either five (for *saa*, *mpx*, and *il1b* primers) or 18 (for *mmp9* and *tnfα* primers) dissected intestines were pooled. RNA was harvested by homogenizing and extracting with Trizol reagent (Invitrogen). Contaminating genomic DNA was eliminated using the Turbo DNA-free kit (Ambion) per manufacturer’s instructions. The RNA (100 ng for *saa*, *mpx*, and *il1b* primers; 320 ng for *mmp9* and *tnfα* primers) was used as templates for generating cDNA with Superscript III Reverse Transcriptase and random primers (Invitrogen) following manufacturer’s instructions. The cDNA was measured in a qPCR reaction with SYBR Fast qPCR master mix (Kapa Biosystems). Assays were performed in triplicate using ABI StepOne Plue RealTime. Data were normalized to *elfa* and analyzed using ΔΔCt analysis. Sequences and annealing temperatures are presented in S1 Table.

### Sample preparation and Illumina sequencing

Dissected intestines were placed in 2-mL screw cap tubes with 0.1 mm zirconia silica beads and 200-μL sterile lysis buffer (20 mM Tris-Cl; 2 mM EDTA; 2.5-mL 20% Tx-100) and frozen in liquid nitrogen. DNA was extracted using Qiamp DNA micro Kit (Qiagen) as detailed in S1 Text. The microbial communities of each sample were characterized by an Illumina HiSeq 2500 Rapid Run (San Diego, CA) sequencing the 16S rRNA gene amplicon by the University of Oregon Genomics and Cell Characterization Facility. The read length was paired-end 150 nucleotide, targeting the V4 region (primers listed in S2 Table). The 16S rRNA gene Illumina reads were clustered using USEARCH 8.1.1803 [64]. The final OTU table was rarefied to a depth of 100,000 (see S2 Data for metadata, S3 Data for OTU taxonomy, and S4 Data for OTU table). Measures of community diversity and similarity (OTU richness, phylogenetic distances, unweighted UniFrac) were calculated in R using *vegan*, *picante*, and *GUniFrac* (See S2 Code). Correlations were calculated in R, and false discovery rate was adjusted using the Benjamini & Hochberg correction in *p*.*adjust* (See S1 Code).

### ENS transplantation

WT donor embryos were labeled by injection of 5% tetramethylrhodamine dextran (3000 MW) at the 1–2 cell stage and reared until the next manipulation in filter-sterilized EM. Embryos at the 12–14 somite stage were mounted in agar, a small hole dissected in the skin, and cells transplanted as previously described [65] and detailed in the S1 Text.

### Statistics

Statistical analysis was performed using Prism (Graphpad software). Statistical significance was defined as *p* < 0.05. Data whose distributions were bounded by 0 were log transformed + 1 prior to statistical analysis. For correlations in Figs 5 and 6, log transformations of neutrophil number and percent OTU were performed so the data met the assumptions of normality and homoscedasticity for linear regression. We note that the relationships and result of multiple linear regression were the same if the data were not log transformed. Throughout, box plots represent the median and interquartile range; whiskers represent the 5–95 percentile. Data for all figures are available in S1 Data.

## Supporting information

S1 Fig *sox10* mutants experience delayed intestinal transit. **(A)** Representative images of wild type (WT, left) and *sox10*^*-*^ (middle and right images) distal intestine. Anti-ElavI labeled enteric neurons are green (green arrow). Scale bar 100 mm. There are no enteric neurons in the *sox10*^*-*^ fish. **(B)** Schematic of the fluorescent food feeding schedule. Color indicates administration of fluorescent tracer; arrow indicates time of imaging for 8 dpf fish. **(C)** Representative images of 8 dpf wild types and *sox10* mutants. Scale bar 100 mm. **(D)** The percent of fish with the indicated fluorescent food color in their intestines at 7 and 8 dpf (i.e. ‘eaters’). Each point represents the percentage of eaters from a separate dish of nine to 30 fish. In total, n > 200 fish per genotype per day. Bars represent mean ± SD. **** p < 0.0001, Student’s T-test.
(TIF)

S2 Fig A higher concentration WT inoculum does not increase intestinal neutrophil number. Inoculating germ-free (GF) fish with a 5x concentrated donor inoculum from WTs does not increase intestinal neutrophil number over what is observed for a 1x concentration. Box plots represent the median and interquartile range, whiskers represent the 5–95 percentile; n ≥ 20; * p < 0.05, ANOVA.
(TIF)

S3 Fig Wild type, *sox10* low neutrophil, and *sox10* high neutrophil intestinal microbial communities are not different based on community wide metrics. **(A)** Bacterial communities in wild type (WT, black closed circles), *sox10*^*-*^ low neutrophil (red closed circles), and *sox10*^*-*^ high neutrophil (red open circles) do not differ based on Canberra distances, as shown in a NMDS analysis. **(B-D)** WT, *sox10*^*-*^ low neutrophil, and *sox10*^*-*^ high neutrophil intestinal microbiota are not different in the number of OTUs present in their communities **(B)**; in phylogenic diversity based on Faith’s PD alpha diversity metric **(C)**; and by pairwise comparisons of unweighted UniFrac distances **(D)**. Box plots represent the median and interquartile range, whiskers represent the 5–95 percentile, n > 30 per group, collected from three independent experiments. **(E)** The average percent abundance of the top 11 representative genera. The ‘other’ group consists of all OTUs that made up less than 0.5% on average in all groups. Error bars represent SEM for the top two most abundant species.
(TIF)

S4 Fig Influence of added *Vibrio* or *E*. *coli* depends on existing microbial community. **(A)** Colonization level of *E*. *coli* HS or *Vibrio* Z20 monoassociated in *sox10*^*-*^ mutants. n > 30. **(B)** The ability of exogenously added bacteria to alter the intestinal neutrophil response depended on the intestinal neutrophil response in the control, which is a result of the microbial community assembled. This graph represents four independent experiments and the difference in intestinal neutrophil influx between the control and the treated samples. The background colors indicate the approximate ranges of neutrophil numbers observed for wild-type germ free fish (WT GF), wild-type conventional fish (WT CV), and conventional *sox10*^*-*^ fish. **(C)** Phylogenetic tree of *Vibrio* and *Escherichia/Shigella* OTUs including the zebrafish isolates *Vibrio* Z20 and *Shewanella* Z12 and *E*. *coli* HS, the representative of the *Escherichia* genus used in experiments. Tree based on 16S sequence. **(D)** A natural zebrafish isolate of *Shewanella* (*Shw* Z12) with a previously demonstrated negative correlation between colonization level and intestinal neutrophil accumulation reduces intestinal neutrophil number in *sox10*^*-*^ mutants through a factor present in the cell free supernatant (CFS). *E*. *coli* HS CFS is not sufficient to reduce neutrophil number. *p < 0.05, ***p < 0.001, ANOVA. Box plots represent the median and interquartile range; whiskers represent the 5–95 percentile.
(TIF)

S1 Table Primers used in qPCR. (DOCX)

S2 Table Primers used for Illumina sequencing. (DOCX)

S1 Text Text file containing expanded materials and methods. (DOCX)

S1 Data Excel spreadsheet containing data plotted in all main and supporting figures. (XLSX)

S2 Data CSV file of meta data containing information about all sequenced samples. (CSV)

S3 Data CSV file of OTU taxonomy of strains identified in sequencing. (CSV)

S4 Data CSV file of percent of each OTU found in each sequenced sample. (CSV)

S1 Code R code for Spearman correlations in Fig 4 and linear regression in Fig 5. (R)

S2 Code R code for measures of community diversity and similarity in S3 Fig. (R)

## Abbreviations

AIC: Akaike’s Information Criterion
CFS: cell-free supernatant
CFU: colony-forming units
CV: conventionally raised
dpf: d post fertilization
ENS: enteric nervous system
FISH: fluorescent in situ hybridization
GF: germ free
GFP: green fluorescent protein
GI: gastrointestinal
HAEC: Hirschsprung-associated enterocolitis
HSCR: Hirschsprung disease
iapMO: intestinal alkaline phosphatase morpholino
IBD: inflammatory bowel disease
OTU: operational taxonomic unit
TNF: tumor necrosis factor
WT: wild-type
