# Supplementary figures and images for: The enteric nervous system promotes intestinal health by constraining microbiota composition
Source: PLoS Biol. 2017 Feb 16;15(2):e2000689. doi: 10.1371/journal.pbio.2000689 (PMC5331947; doi:10.1371/journal.pbio.2000689)

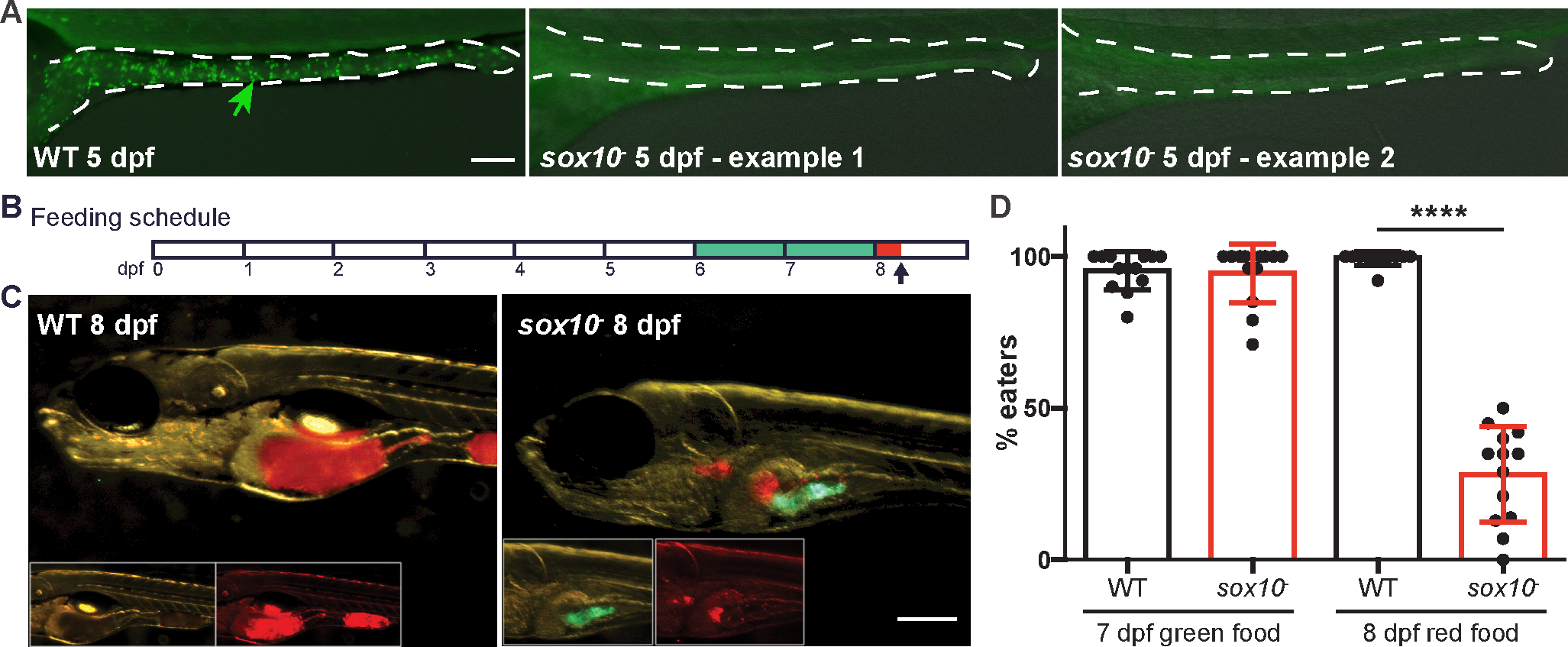

Supplement: S1 Fig — (A) Representative images of wild type (WT, left) and sox10- (middle and right images) distal intestine. Anti-ElavI labeled enteric neurons are green (green arrow). Scale bar 100 mm. There are no enteric neurons in the sox10- fish. (B) Schematic of the fluorescent food feeding schedule. Color indicates administration of fluorescent tracer; arrow indicates time of imaging for 8 dpf fish. (C) Representative images of 8 dpf wild types and sox10 mutants. Scale bar 100 mm. (D) The percent of fish with the indicated fluorescent food color in their intestines at 7 and 8 dpf (i.e. ‘eaters’). Each point represents the percentage of eaters from a separate dish of nine to 30 fish. In total, n > 200 fish per genotype per day. Bars represent mean ± SD. **** p < 0.0001, Student’s T-test. (TIF) [file pbio.2000689.s001.tif]

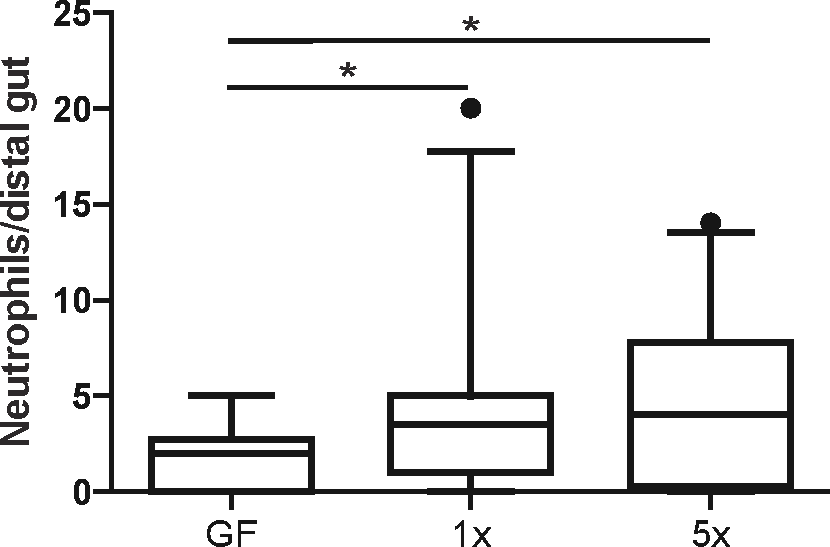

Supplement: S2 Fig — Inoculating germ-free (GF) fish with a 5x concentrated donor inoculum from WTs does not increase intestinal neutrophil number over what is observed for a 1x concentration. Box plots represent the median and interquartile range, whiskers represent the 5–95 percentile; n ≥ 20; * p < 0.05, ANOVA. (TIF) [file pbio.2000689.s002.tif]

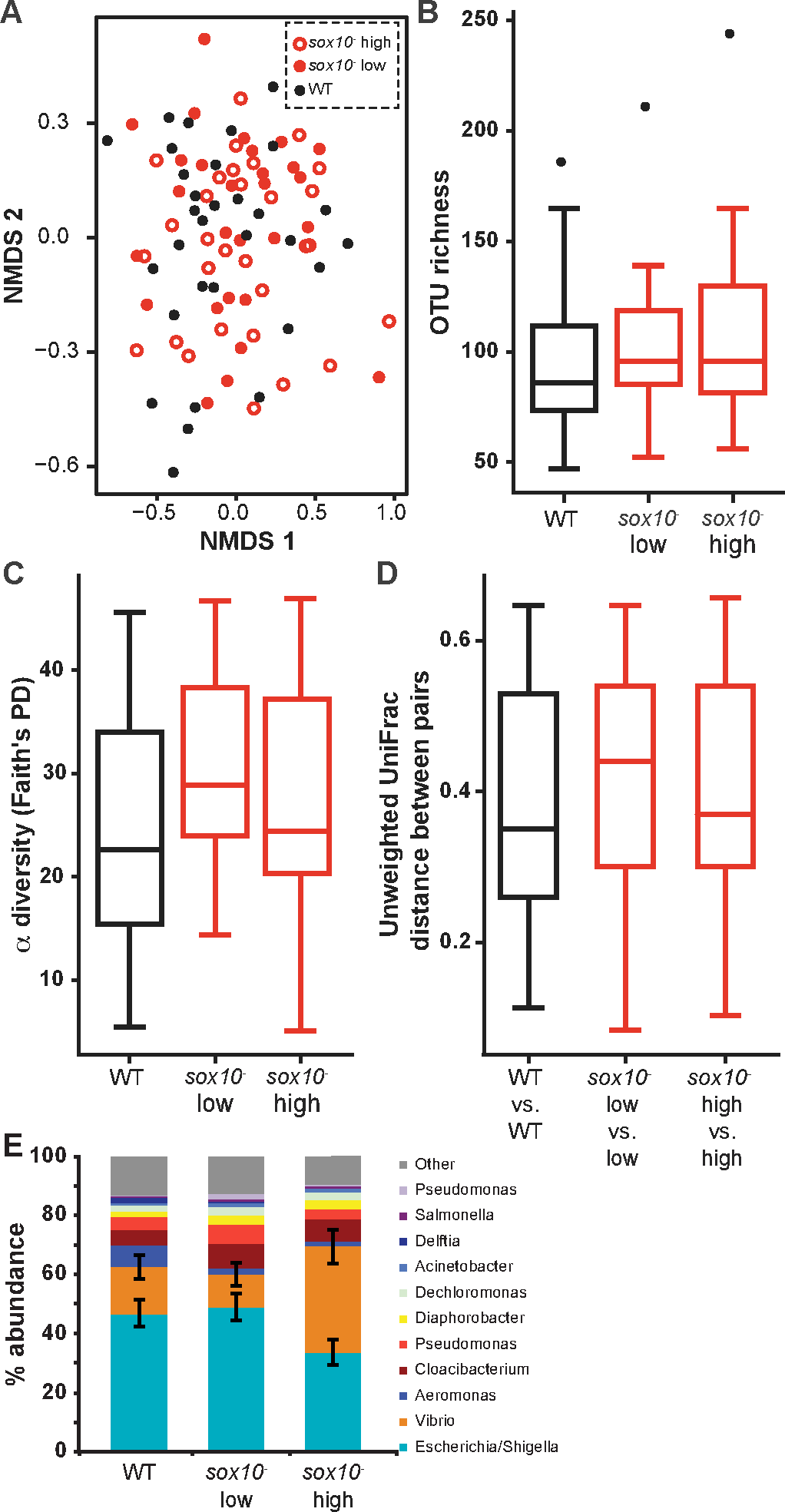

Supplement: S3 Fig — (A) Bacterial communities in wild type (WT, black closed circles), sox10- low neutrophil (red closed circles), and sox10- high neutrophil (red open circles) do not differ based on Canberra distances, as shown in a NMDS analysis. (B-D) WT, sox10- low neutrophil, and sox10- high neutrophil intestinal microbiota are not different in the number of OTUs present in their communities (B); in phylogenic diversity based on Faith’s PD alpha diversity metric (C); and by pairwise comparisons of unweighted UniFrac distances (D). Box plots represent the median and interquartile range, whiskers represent the 5–95 percentile, n > 30 per group, collected from three independent experiments. (E) The average percent abundance of the top 11 representative genera. The ‘other’ group consists of all OTUs that made up less than 0.5% on average in all groups. Error bars represent SEM for the top two most abundant species. (TIF) [file pbio.2000689.s003.tif]

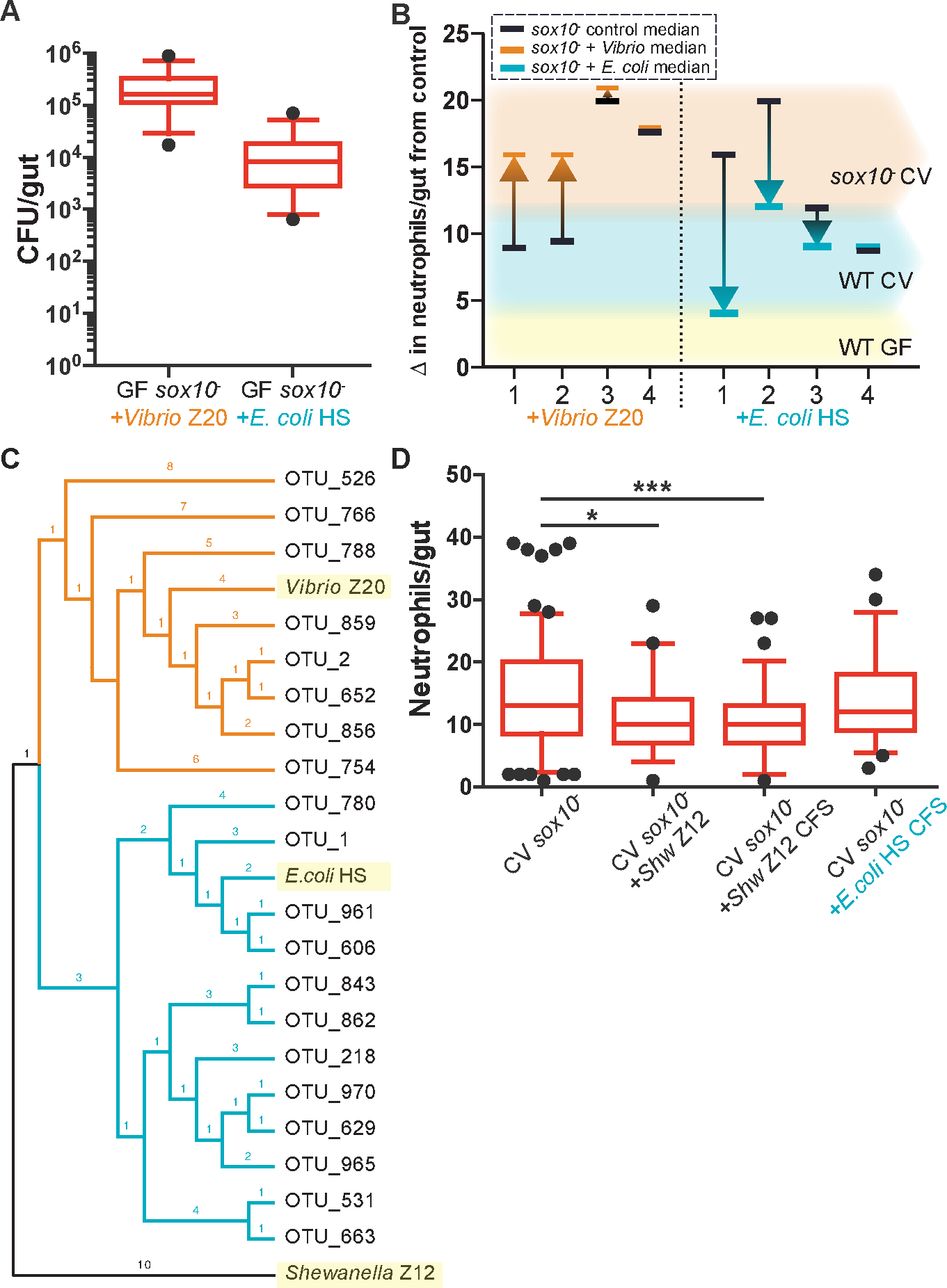

Supplement: S4 Fig — (A) Colonization level of E. coli HS or Vibrio Z20 monoassociated in sox10- mutants. n > 30. (B) The ability of exogenously added bacteria to alter the intestinal neutrophil response depended on the intestinal neutrophil response in the control, which is a result of the microbial community assembled. This graph represents four independent experiments and the difference in intestinal neutrophil influx between the control and the treated samples. The background colors indicate the approximate ranges of neutrophil numbers observed for wild-type germ free fish (WT GF), wild-type conventional fish (WT CV), and conventional sox10- fish. (C) Phylogenetic tree of Vibrio and Escherichia/Shigella OTUs including the zebrafish isolates Vibrio Z20 and Shewanella Z12 and E. coli HS, the representative of the Escherichia genus used in experiments. Tree based on 16S sequence. (D) A natural zebrafish isolate of Shewanella (Shw Z12) with a previously demonstrated negative correlation between colonization level and intestinal neutrophil accumulation reduces intestinal neutrophil number in sox10- mutants through a factor present in the cell free supernatant (CFS). E. coli HS CFS is not sufficient to reduce neutrophil number. *p < 0.05, ***p < 0.001, ANOVA. Box plots represent the median and interquartile range; whiskers represent the 5–95 percentile. (TIF) [file pbio.2000689.s004.tif]
