# Supplementary material for: The enteric nervous system promotes intestinal health by constraining microbiota composition
Source: PLoS Biol. 2017 Feb 16;15(2):e2000689. doi: 10.1371/journal.pbio.2000689 (PMC5331947; doi:10.1371/journal.pbio.2000689)
Supplement: S1 Table — (DOCX) [file pbio.2000689.s005.docx]

**Table S1. Primers used in qPCR (related to Fig 3)**

| **Target** | **Primer sequence** | **Annealing temp.** | **Reference** |
| --- | --- | --- | --- |
| *elfa* | 5’ cttctcaggctgactgtgc | 58 or 62 | McCurley, et al. |
|  | 5’ ccgctagcattaccctcc |  |  |
| *mpx* | 5’ TCGAGATCAAAAGCTGGGATA | 58 |  |
|  | 5’ CCGAGATGGCGATAGGTTG |  |  |
| *saa* | 5’ CGGGGTCCTGGGGGCTATTG | 58 |  |
|  | 5’ GTTGGGGTCTCCGCCGTTTC |  |  |
| *il1b* | 5’ CATCAAACCCCAATCCACAG | 58 |  |
|  | 5’ CACCACGTTCACTTCACGCT |  |  |
| *c3* | 5’ CGGACGCTGACATCTACCAA | 58 |  |
|  | 5’ TCCAGGTCTGCTCTCCCAAG |  |  |
| *tnfα* | 5’ GCGCTTTTCTGAATCCTACG | 62 |  |
|  | 5’ TGCCCAGTCTGTCTCCTTCT |  |  |
| *mmp9* | 5’ gctgctcatgagtttggaca | 62 | Marjoram, L et al. |
|  | 5’ agggccagttctaggtcctt |  |  |
