# Supplementary material for: The enteric nervous system promotes intestinal health by constraining microbiota composition
Source: PLoS Biol. 2017 Feb 16;15(2):e2000689. doi: 10.1371/journal.pbio.2000689 (PMC5331947; doi:10.1371/journal.pbio.2000689)
Supplement: S2 Table — (DOCX) [file pbio.2000689.s006.docx]

**Table S2. Primers used for Illumina sequencing (related to Figure 4 and S3)**

| **Oligo Name** | **Sequence** |
| --- | --- |
| 806-R_TCACCTAG | CAAGCAGAAGACGGCATACGAGATtcacctagAGTCAGTCAGCCGGACTACHVGGGTWTCTAAT |
| 806-R_CAAGTCGT | CAAGCAGAAGACGGCATACGAGATcaagtcgtAGTCAGTCAGCCGGACTACHVGGGTWTCTAAT |
| 806-R_CTGTATGC | CAAGCAGAAGACGGCATACGAGATctgtatgcAGTCAGTCAGCCGGACTACHVGGGTWTCTAAT |
| 806-R_AGTTCGCA | CAAGCAGAAGACGGCATACGAGATagttcgcaAGTCAGTCAGCCGGACTACHVGGGTWTCTAAT |
| 806-R_ATCGGAGA | CAAGCAGAAGACGGCATACGAGATatcggagaAGTCAGTCAGCCGGACTACHVGGGTWTCTAAT |
| 806-R_AAGTCCTC | CAAGCAGAAGACGGCATACGAGATaagtcctcAGTCAGTCAGCCGGACTACHVGGGTWTCTAAT |
| 806-R_TGGATGGT | CAAGCAGAAGACGGCATACGAGATtggatggtAGTCAGTCAGCCGGACTACHVGGGTWTCTAAT |
| 806-R_AGGTGTTG | CAAGCAGAAGACGGCATACGAGATaggtgttgAGTCAGTCAGCCGGACTACHVGGGTWTCTAAT |
| 806-R_GACGAACT | CAAGCAGAAGACGGCATACGAGATgacgaactAGTCAGTCAGCCGGACTACHVGGGTWTCTAAT |
| 806-R_GTTCTTCG | CAAGCAGAAGACGGCATACGAGATgttcttcgAGTCAGTCAGCCGGACTACHVGGGTWTCTAAT |
| 806-R_TTCGCCAT | CAAGCAGAAGACGGCATACGAGATttcgccatAGTCAGTCAGCCGGACTACHVGGGTWTCTAAT |
| 806-R_CAACTCCA | CAAGCAGAAGACGGCATACGAGATcaactccaAGTCAGTCAGCCGGACTACHVGGGTWTCTAAT |
| 515-F_GACACAGT | AATGATACGGCGACCACCGAGATCTACACgacacagtTATGGTAATTGTGTGCCAGCMGCCGCGGTAA |
| 515-F_GCATAACG | AATGATACGGCGACCACCGAGATCTACACgcataacgTATGGTAATTGTGTGCCAGCMGCCGCGGTAA |
| 515-F_ACAGAGGT | AATGATACGGCGACCACCGAGATCTACACacagaggtTATGGTAATTGTGTGCCAGCMGCCGCGGTAA |
| 515-F_CCACTAAG | AATGATACGGCGACCACCGAGATCTACACccactaagTATGGTAATTGTGTGCCAGCMGCCGCGGTAA |
| 515-F_TGTTCCGT | AATGATACGGCGACCACCGAGATCTACACtgttccgtTATGGTAATTGTGTGCCAGCMGCCGCGGTAA |
| 515-F_GATACCTG | AATGATACGGCGACCACCGAGATCTACACgatacctgTATGGTAATTGTGTGCCAGCMGCCGCGGTAA |
| 515-F_AGCCGTAA | AATGATACGGCGACCACCGAGATCTACACagccgtaaTATGGTAATTGTGTGCCAGCMGCCGCGGTAA |
| 515-F_CTCCTGAA | AATGATACGGCGACCACCGAGATCTACACctcctgaaTATGGTAATTGTGTGCCAGCMGCCGCGGTAA |
