# Supplementary material for: The enteric nervous system promotes intestinal health by constraining microbiota composition
Source: PLoS Biol. 2017 Feb 16;15(2):e2000689. doi: 10.1371/journal.pbio.2000689 (PMC5331947; doi:10.1371/journal.pbio.2000689)
Supplement: S1 Text — (DOCX) [file pbio.2000689.s007.docx]

**Supplemental materials and methods**

*Food transit assay*

Fluorescent green and red food was prepared as described previously (Field, Kelley, Martell, Goldstein, & Serluca, 2009). Fertilized eggs from four sets of *sox10^+/-^* parents were collected. Clutches were sorted into petri dishes with 25 WT siblings and 25 *sox10* mutant siblings, resulting in 13 petri dishes. In the morning on 6 and 7 dpf, approximately 2 mg of prepared fluorescent green food was administered per petri dish. In the afternoon on 7 dpf, larvae were assessed for the presence of green fluorescent food in their intestines. All larvae were then moved to a new petri dish with 2 mg of prepared fluorescent red food, and examined for the presence of red fluorescent food in their intestines in the morning on 8 dpf.

*Histology and neutrophil analysis*

Zebrafish larvae were fixed in 4% paraformaldehyde (PFA) overnight. Whole larvae were stained with Myeloperoxidase kit (Sigma) following the manufacturer’s protocol. Following staining, larvae were washed in 1× PBS, embedded in paraffin, and approximately 7 μm thick sections were cut and mounted on glass slides. Cells with strong MPO staining in the intestinal epithelium were quantified in 20 serial sections rostral to the anus, corresponding to the distal 140 μm of the intestine. For analysis of neutrophils in *mpx:GFP* fish, the fish were anesthetized in Tricaine (Western Chemical, Inc.) and mounted in 4% methylcellulose (Fisher). Subsequently, their intestines were sterilely dissected and GFP-positive cells in the intestine (not including the bulb) were quantified visually for each fish using a fluorescent microscope (SteREO Discovery.V8, Zeiss).

*Quantification of proliferating cells*

Larvae were immersed in 100 μg/ml EdU (A10044, Invitrogen) for 16 h. Subsequently, larvae were fixed in 4% PFA for 4 h at room temperature with gentle shaking, processed for paraffin embedding, and cut into 7 μm sections. Slides were processed according to manufacturers instructions for the Click-iT EdU Cell Proliferation Assay Kit (C35002, Molecular Probes). EdU-labeled nuclei within the intestinal epithelium were counted over 30 serial sections beginning at the esophageal-intestinal junction and proceeding caudally to the bulb.

*Fluorescent in situ hybridization*

Tissue sections were incubated with a hybridization buffer (pre-warmed; 20 mM Tris-HCL pH 7.4, 0.9 M NaCl, 0.1% SDS, 35% Formamide) containing three oligonucleotide probes at a concentration of 0.25 pmol/μl each. To visualize all bacteria, we used a mixture of probes: Eub338-I (GCTGCCTCCCGTAGGAGT), Eub338-II (GCAGCCACCCGTAGGTGT), and Eub338-III (GCTGCCACCCGTAGGTGT) (Integrated DNA Technologies) (Loy, Maixner, Wagner, & Horn, 2007).

*ENS transplantation*

Wild-type donor embryos were labeled by injection of 5% tetramethylrhodamine dextran (3000 MW) at the 1-2 cell stage and reared until the next manipulation in filter-sterilized EM. Embryos at the 12-14 somite stage were mounted in agar, a small hole dissected in the skin, and cells transplanted. Briefly, approximately 10-20 vagal neural crest cells were aspirated from the region just posterior of the developing ear of labeled wild-type donors and transplanted to the same region of unlabeled hosts derived from an incross of *sox10*^+/-^ parents. Following transplantation, both donors and hosts were maintained for 4 h in zebrafish physiological saline containing Penicillin-Streptomycin (Pen-Strep; Sigma), which allowed the dissected skin to heal. Because hosts were maintained first in filter-sterilized embryo medium and then in filter-sterilized physiological saline containing Pen-Strep, they never acquired a microbiota. Therefore, beginning at 4 h after transplantation, host embryos were co-housed with unmanipulated controls that had been previously reared in fish water, at a ratio of 12 hosts to 20 controls, in a 100 × 20 mm petri plate containing EM. Hosts were segregated from controls by a mesh enclosure. At 8 dpf, hosts were fixed in 1× formaldehyde/1× PBS for 3 h at room temperature, washed 3 × 10 min in 1× PBS. Whole larvae were stained with Myeloperoxidase (MPO) kit (Sigma) following the manufacturer’s protocol and subsequently we performed immunohistochemistry with anti-Elavl (Kuhlman & Eisen, 2007) to reveal enteric neurons. MPO+ neutrophils were quantified in whole-mount larvae along the length of the gut.

*Sample preparation for Illumina sequencing*

Frozen samples were thawed quickly at 65 °C and subjected to mechanical disruption using a Bullet Blender (Next Advance) for 1 min 15 sec on a setting of 4. Ten μL Proteinase K was added to each sample followed by 200 μL buffer AL, subsequently samples were incubated at 56 °C for 30 min. Then 200 μL 100% EtOH was added to each sample. All liquid was then added to the column and centrifuged. After two washes, samples were eluted in 25 μL sterile, PCR water. Sample concentration was quantified using the Qubit (Thermo Fisher) with a range of resulting concentrations from 0.5 mg/mL to 6.8 mg/mL.

**Supplemental references**

Field, H. a., Kelley, K. a., Martell, L., Goldstein, a. M., & Serluca, F. C. (2009). Analysis of gastrointestinal physiology using a novel intestinal transit assay in zebrafish. *Neurogastroenterology & Motility*, *21*(3), 304–312. doi:10.1111/j.1365-2982.2008.01234.x

Kuhlman, J., & Eisen, J. S. (2007). Genetic screen for mutations affecting development and function of the enteric nervous system. *Developmental Dynamics*, *236*(1), 118–127. doi:10.1002/dvdy.21033

Loy, a., Maixner, F., Wagner, M., & Horn, M. (2007). probeBase--an online resource for rRNA-targeted oligonucleotide probes: new features 2007. *Nucleic Acids Research*, *35*(Database), D800–D804. doi:10.1093/nar/gkl856
